# eIF6 as a Promising Diagnostic and Prognostic Biomarker for Poorer Survival of Cutaneous Melanoma

**DOI:** 10.3389/fonc.2022.848346

**Published:** 2022-05-30

**Authors:** Fangyingnan Zhang, Saquib Waheed, Ubaldo Armato, Jun Wu, Chao Zhang, Zhibin Li

**Affiliations:** ^1^ School of Biomedical Engineering, Sun Yat-sen University, Guangzhou, China; ^2^ Department of Burn and Plastic Surgery, Shenzhen Institute of Translational Medicine, The First Affiliated Hospital of Shenzhen University, Shenzhen Second People’s Hospital, Shenzhen, China

**Keywords:** eIF6, melanoma, diagnostic and prognostic, biomarker, tumor therapy

## Abstract

**Background:**

Skin cutaneous melanoma (SKCM) is the deadliest skin cancer and has the most rapidly increasing incidences among all cancer types. Previous research elucidated that melanoma can only be successfully treated with surgical abscission in the early stage. Therefore, reliable and specific biomarkers are crucial to melanoma diagnosis since it often looks like nevi in the clinical manifestations. Moreover, identifying key genes contributing to melanoma progression is also highly regarded as a potential strategy for melanoma therapy. In this respect, translation initiator eIF6 has been proved as a pro-tumor factor in several cancers. However, the role of eIF6 in the skin cutaneous melanoma progression and its potential as a prognostic marker is still unexplored.

**Methods:**

The immunochemical analysis of clinical specimens were served to assess eIF6 expression levels. Gene Expression Profiling Interactive Analysis (GEPIA) database consultations allowed us to find the survival rates of the eIF6-overexpressed patients. eIF6 cellular effects were evaluated in an eIF6-overexpressed A375 cell line constructed with a lentivirus. The analysis of down-stream effectors or pathways was conducted using C-Bioportal and STRING databases.

**Results:**

Our results revealed that eIF6 was highly over-expressed in melanomas compared to normal skin specimens, and thus the abnormally high level of eIF6 can be a diagnostic marker for melanoma. The in silica analysis indicated that patients with eIF6 over-expression had lower survival rates than that low-expression in SKCM. Meanwhile, similar results also could be found in the other four types of cancers. *In vitro*, over-expression of eIF6 increased the proliferation and migration of melanoma cells. Correspondingly, pan-cancer clustering analysis indicated the expression level of intermediate filament proteins was correlated with that of eIF6 expression. In our study, all over-expressed keratin proteins, in accordance with over-expressed eIF6, had a negative correlation with melanoma prognosis. Moreover, the decreased methylation level of keratin genes suggested a new potential regulation mode of eIF6.

**Conclusions:**

The up-regulated eIF6 could be a potential diagnostic and prognostic biomarker of melanoma. This study also provides insights into the potential role of eIF6 in pan-cancer epigenetic regulation.

## Introduction

Melanoma, also called malignant melanoma, is a type of skin cancer that arises from pigment-producing cells called melanocytes. It accounts for 10% of newly diagnosed cases of overall skin cancers and further increases in its prevalence and mortality worldwide. To date, the skin melanoma incidence rate has increased five-fold since the mid-1980s (1, 2). Nowadays, melanoma has become the most lethal type of skin cancer, with a mortality rate second behind lung cancer (3). The ideal treatment of melanoma is through surgical resection at the early stage. Otherwise, the survival rate of patients may be decreased significantly when the metastatic dissemination is occurred (4). Therefore, a precise early diagnosis is pivotal to the good prognosis of melanoma. Especially, the clinical manifestations of melanoma are not obvious in the early stage, mostly present as nevi-like skin lesions, which may or may not be associated with ulceration or bleeding. Thus, the clinical diagnoses are frequently unreliable (5, 6). In recent years, newly produced monoclonal antibodies specifically target tumor-associated antigens enable researchers to detect the onset and recurrence of malignant melanomas and make a specific histopathological diagnosis. Actually, monoclonal antibodies (McAbs) are developed for the histopathological diagnosis and classification of the cancers, such as the HMSA1 and HMSA2 McAbs that targeted melanosome-associated antigens. Nevertheless, the specificity of these McAbs were far from satisfaction. Some more specific McAbs, including NK1C3, S-100 and HMB-45, have been developed recently (7–9) to address this shortcoming. However, melanoma shows significant heterogeneity. In clinical cancer diagnosis, the cases with Melan-A negative or even with S100 negative have been often reported (10, 11). Therefore, the researchers hope to seek more specific diagnostic biomarkers to avoid misdiagnosis cases. Meanwhile, drug and immune therapy are the main choices for metastatic melanoma patients. Target therapy with BRAF/MEK inhibitors in metastatic melanoma has shown a high response rate. However, the cases that have resistance to this treatment still frequently occur due to the unsatisfactory selectivity of chemotherapeutic agents. Hence, there is an urgency to seek more promising diagnostic and prognostic biomarkers for melanoma therapy.

Previous reports showed that the continuous proliferating melanoma cells demand a high level of protein synthesis, and the dysregulation of mRNA translation is generally regarded as a typical tumorigenesis feature of melanoma (12, 13). In this process, protein synthesis includes four steps: initiation, elongation, termination, and ribosome recycling. The initiation is the most important step of protein translation because it is both highly regulated and rate-limited. In this respect, a set of proteins named eukaryotic Initiation Factors (eIFs) control the onset of translation in eukaryotic cells (14). Dozens of researches have identified the cancerous function in different eIFs. For example, recent studies have proved that eIF4B contributes to the cellular adaptation of asparagine in BRAF-mutated A375 melanoma. Meanwhile, in prostate cancer cells, eIF5B can activate the PD-1 checkpoint of the T cells by interacting with Wig1, causing T cell exhaustion and promoting tumor development and metastasis (15, 16). Among eIFs, eIF6 has attracted enormous interest because it not only regulates the ribosomal 60S subunit genesis inside the nucleus but also mediates ribosomal assembly in the cytoplasm (17). In 2008, Biffo et al. have firstly proved eIF6 as a rate-limiting factor in cell-cycle and tumorigenesis. Nowadays, the tumor-promoting pathways associated with eIF6 have been found in various types of cancer cells (18). For instance, in the myc-induced lymphomas mice model, eIF6 impairment can significantly reduce the tumor growth and prolong the tumor-free survival time through an mTORC-independent mechanism (19). In contrast, previous research found over-expression of eIF6 in ovarian cells and melanoma cell lines can effectively increase cell mobility and proliferation *via* CDC42 up-regulation (20). Furthermore, the increased eIF6 level has been reported to play a major role in association with poor prognostication of colorectal cancer, non-small cell lung carcinoma and malignant pleural mesothelioma (21–24). Thus, eIF6 is a promising diagnostic and prognostic candidate in melanoma.

In this work, we investigated the eIF6 expression features and its role in melanoma progression using clinical specimens and the TCGA database. We examined the prognostic value of eIF6 according to its expression and analyzed the patients’ survival data to infer its potential melanoma-promoting mechanisms. Our results revealed that the high eIF6 expression accompanied more dynamic cell skeleton gene expression and led to accelerated cellular proliferation. These findings elucidated the underlying regulation mechanisms of eIF6 in melanoma, and our pan-cancer analysis also provided clues of an epigenetic function of eIF6 in other types of cancers.

## Methods

### Gene Expression Profiling Interactive Analysis (GEPIA)

The analysis of patients’ survival rates was conducted using GEPIA, a web tool based on TCGA and GTEx databases. Based on the RNA-sequencing results, GEPIA supplies the expression levels of specific genes in various cancer types compared to those of adjacent normal tissues. GEPIA divides the cancer clinical data into two groups and compares the prognosis based upon the expression levels of the gene of interest. GEPIA is available at http://gepia.cancer-pku.cn/ (25).

### c-BioPortal Analysis

The c-Bio Cancer Genomics Portal (http://cbioportal.org) is an open-source online platform supplying a multidimensional view of cancer genomics data. By now, it holds the data from 225 cancer studies. We classified the SKCM samples into an eIF6 overexpressing (i.e., an “altered”) group and an “unaltered” group. We compared the two groups’ RNA-sequencing data to assess the differences in expressed genes and DNA methylation data. We analyzed the altered group samples in the “TCGA Firehouse Legacy” dataset, which holds data from 479 skin melanoma samples. The search parameters of the altered group were “mRNA expression Z-scores relative to diploid samples”. The Z-score threshold was 2, which descripted the variation level of a certain number in samples identification. Since these samples accounted for 14% in all the SKCM patients, the 14% top of eIF6 expressed samples were defined as the “altered” group in SKCM patients (26).

### Database for Annotation, Visualization, and Integration Discovery (DAVID) Analysis

We used DAVID to make the annotation and KEGG analysis. Resources in DAVID aim to interpret gene function from an extensive list. DAVID is also capable for KEGG pathway enrichment analysis. We got the list of differentially expressed genes (DEGs) from the c-BioPortal and gave functional annotations using DAVID. The DAVID is available at http://david.niaid.nih.gov (27).

### Protein-Protein Interaction Analysis

The Search Tool for Recurring Instances of Neighboring Genes (STRING) database can visualize protein-protein interactions by presenting genes as colored nodes and linking the interacting genes with lines. In the interaction map of STRING, the genes which function or bind closely occupy neighboring places and have thick lines linking each other. STRING is also capable for gene annotation enrichment analysis, classifying the genes by the Gene Ontology terms. The STRING database is available at https://string-db.org/ (28).

### Clinical Specimens and Immunochemistry

The First Affiliated Hospital of Shenzhen University provided us with melanoma samples. Immunochemistry experiments were conducted as described in the previous research (29). The antibodies used were purchased from Cell Signaling Technology, Inc. (eIF6, 3263S; HMB45, 38815S; S100, 90393) and Boster Biological Technology Co., Ltd. (Melan-A, M02033).

### Cell Lines and Vectors

The A375 cell line was obtained from the American Type Culture Collection (ATCC). The cells were cultured in Dulbecco’s Modified Eagle Medium (Bibico, 11965084) containing 10% fetal bovine serum (Gibico, 10099) and 1% Penicillin-Streptomycin (Gibico, 15140122). The eIF6 over-expression cell line was established by infecting A375 cells with lentivirus, and the counterpart GFP-expression A375 cells as the control group. The target genes were carried by pGWLV01 plasmids (bought from GENEWIZ Cooperation). The plasmids were transfected into 293T cells with the help of polyethylenimine to produce virus. The virus was harvested at 48 and 72 h post-transfection and A375 cells were infected in the presence of 10 μg/mL of polybrene and 10 mM HEPES. The infected cells were screened by treatment with puromycin (50 μg/mL) for two days.

### Wound Healing Assay

The wound-healing assay was used to test the ability of the cells migration as previously reported (30). Briefly, 2×10^6^ cells were plated onto a 100-mm dish to create a confluent monolayer. The cells were scratched and resulting in a straight wound. The wound width was measured after incubation for 24 and 48 h.

### Statistical Analysis

The significance test of change was evaluated with *P* value. *P* value < 0.05 was labeled as “*”. *P*-value < 0.01 was labeled as “**”. *P*-value < 0.001 was labeled as “***”.

## Results

### eIF6 Is Up-Regulated in Skin Melanomas and Is Related to Poor Prognosis

**Figure 1:**
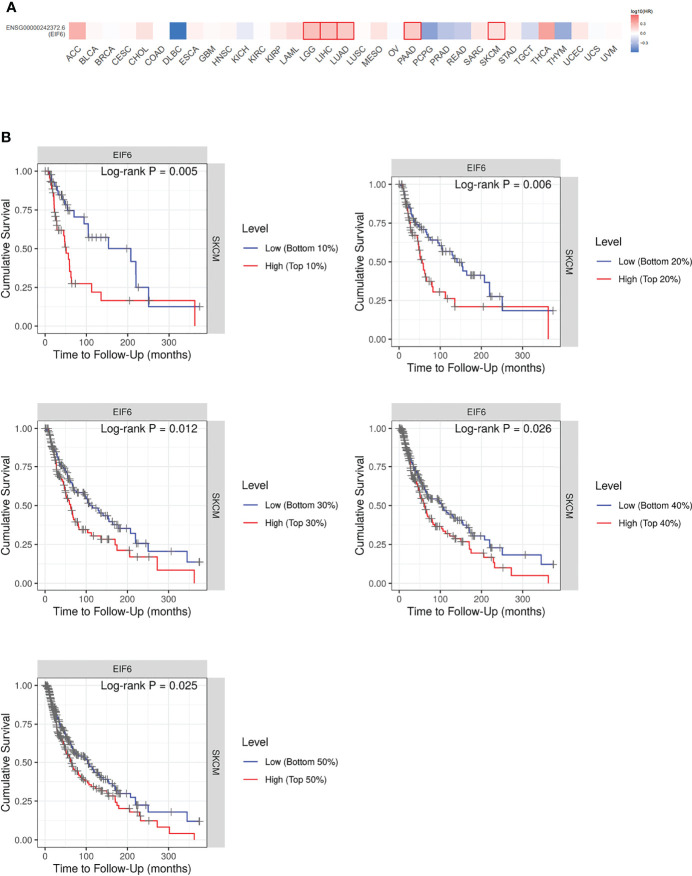
Analysis of the correlation between cancer survival rates and eIF6 expression level. **(A)** Survival analysis of eIF6 in 33 types of cancers: The survival rates of 33 types of cancers were analyzed. The cancer types whose eIF6-high expressed patients showed significantly poorer prognoses were labeled with red frame. The cancer types whose survival rates were improved by high eIF6 levels were labeled with blue. **(B)** Melanoma patients were grouped according to their eIF6 expression levels, and their respective survival rates were compared.

To identify the impact of eIF6 on tumor progression. We first compared the survival rate of 33 common types of cancers during the up-regulation of eIF6. Among them, about 50% of the cancers’ survival rates were lower in the eIF6 higher expression group. The patients with low-eIF6 expression survived longer than that of eIF6 over-expression in seven types of cancers. In eIF6 over-expression specimens, the survival rates of brain lower-grade glioma (LGG), liver hepatocellular carcinoma (LIHC), lung adenocarcinoma (LUAD), pancreatic adenocarcinoma (PAAD), and skin cutaneous melanoma (SKCM) were all significantly reduced (**Figure 1A**).

Among these five types of cancers, the eIF6’s impact on LGG, LIHC and LUAD progression had been reported previously, while that on melanoma was still unclear. Thus we focused our interests on the melanoma study. We compared the survival curves of melanoma patients with high or low eIF6 expression levels and grouped them according to gradient inclusion criteria. For instance, we compared the top 10% of high-eIF6 expression patients with the bottom 10% of low-eIF6 expression patients. Then, the top 20% of patients were compared with the bottom 20%. In all of the survival curves, melanoma patients with high eIF6 expression had worse prognoses (**Figure 1B**).

**Figure 2:**
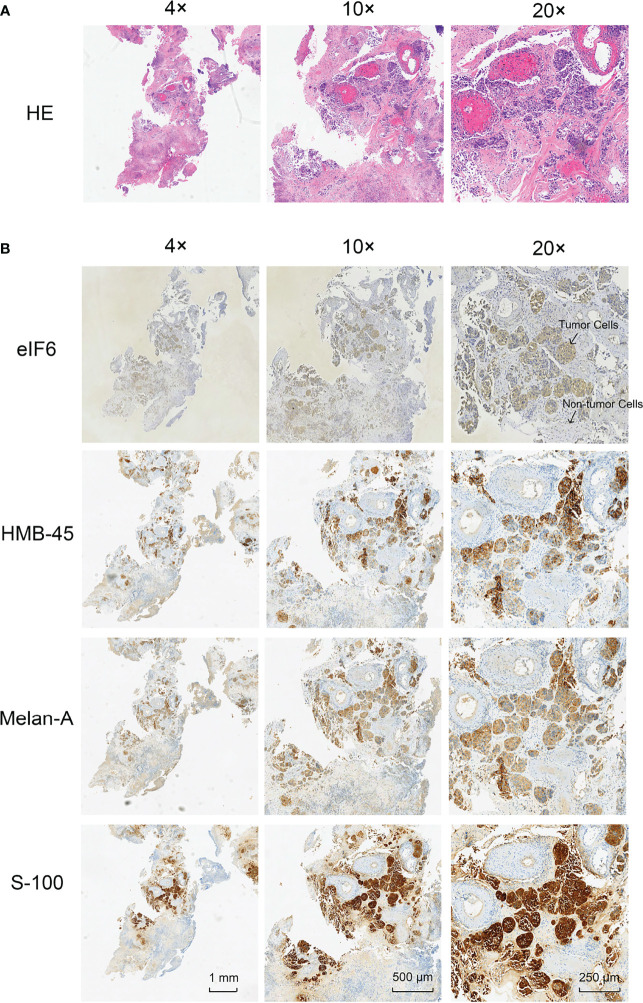
Histological examination of the tumor specimens (Area size: 0.5 cm^2^). **(A)** Hematoxylin-eosin staining (H&E) shows the histology of melanoma tumor. **(B)** Immunohistochemical staining of eIF6, HMB-45, Melan-A, and S-100 in melanoma tumor slices, respectively.

Immunochemistry was used to determine the level of eIF6 protein in the melanoma specimens. Hematoxylin and eosin (HE) staining and immunochemistry analysis of HMB-45, S-100, and Melan-A were used to identify melanoma cells. The results indicated that melanoma cells had higher levels of eIF6 expression when compared to adjacent normal tissues (**Figure 2**). The eIF6 expression through all the stages of melanoma development was further investigated by GEPIA analysis, the results showed that compared with the stage 0, eIF6 level was up-regulated from stage I to stage IV. Especially in stage I and stage II, eIF6 level was even higher than that in the later stages (**Figure S2**). Additionally, the up-regulated eIF6 expression was further confirmed by Timer analysis, which suggest the potential effectiveness of eIF6 in early stage melanoma diagnosis (**Figure S3**).

### eIF6 Promotes the Proliferation and Migration of Melanoma Cell Lines

The poor survival rate of the eIF6 high expression group suggested that eIF6 profoundly impacted the tumor cells. As a translation initiation factor, eIF6 expression is intimately linked to ribosome biogenesis and thus affects protein synthesis. It is possible that eIF6 acts as a rate-limiting factor in cell proliferation. The cellular function of eIF6 was investigated using the melanoma cell line A375. eIF6 was over-expressed with lentivirus, and the over-expressed GFP cells were used as control.

**Figure 3:**
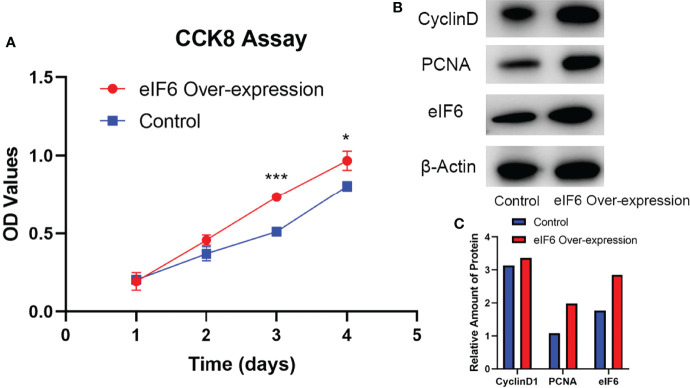
Evaluation of the cellular functions of high-expressed eIF6 in A375 melanoma cells. **(A)** Growth curves comparison between eIF6-overexpressing and wild type (control) A375 cells. The growth rate of eIF6 overexpressing cells was significantly higher than controls. **(B)** Western blot analysis confirms the correlation of up-regulated eIF6 with the cell proliferation marker CyclinD1 and PCNA. **(C)** Quantification of the Western blot results by Image J. The grey density of the bands was measured by Image J, and the target gene expression level was normalized with the grey density of β-actin. The significance test of change was evaluated with P value. P value < 0.05 was labeled as “*”. P-value < 0.01 was labeled as “**”. P-value < 0.001 was labeled as “***”.

While eIF6 was over-expressed, the growth curves of cells showed a significant shifted on day three and four (**Figure 3A**). We examined the cell-cycle markers PCNA and Cyclin D1 to validate the accelerated growth rates of cells. As shown in **Figures 3B, C**, all of these proliferation markers showed a drastic up-regulation accompanied accelerated cell growth phenotypes when eIF6 was over-expressed.

**Figure 4:**
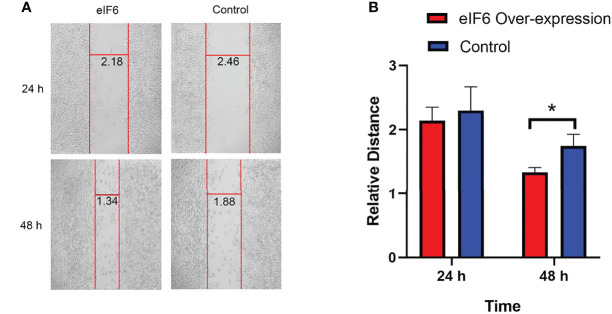
Wound healing assay measures the migration of melanoma cells with over-expression of eIF6. **(A)** The vertical red lines show the wound edges at 24 h and 48 h after scratching, and the horizontal lines show the relative distances between red stripes at the same observation times. The eIF6 over-expression cells migrated more quickly than the control group. **(B)** The spaces between wound edges were measured, and the values were significantly lesser than the eIF6 over-expression group after incubation for 48 h. The significance test of change was evaluated with P value. P value < 0.05 was labeled as “*”. P-value < 0.01 was labeled as “**”. P-value < 0.001 was labeled as “***”.

At both 24 and 48 hours, cells migration ability was assessed during the wound healing. We used Image J software to measure the average width of the scratches. As shown in **Figure 4**, after 24-hours incubation, the eIF6 over-expressed group was 10% narrower than the control group. It reached 25% at 48-hours, indicating over-expression of eIF6 could accelerate the migration of malignant melanoma cells *in vitro*.

### Upregulation of Ribosomal Proteins and Intermediate Filaments Is Linked to High eIF6 Expression

Several theories have been proposed to explain how eIF6 contributes to cell proliferation and migration, but none has been accepted as the most plausible. Using high-throughput sequencing data, we investigated the potential downstream effectors of eIF6. The results demonstrated that eIF6 expression significantly affected the survival rates of SKCM, LGG, LIHC, PAAD, and LUAD cells. In addition, we clustered the differentially expressed genes between the eIF6 high expression and low expression groups in these five types of cancers (the list of differential genes is in **Supplementary Table 1**). We found that the ribosomal genes showed an increased mRNA level in the eIF6 high expression group due to the co-expression of ribosomal genes.

**Figure 5:**
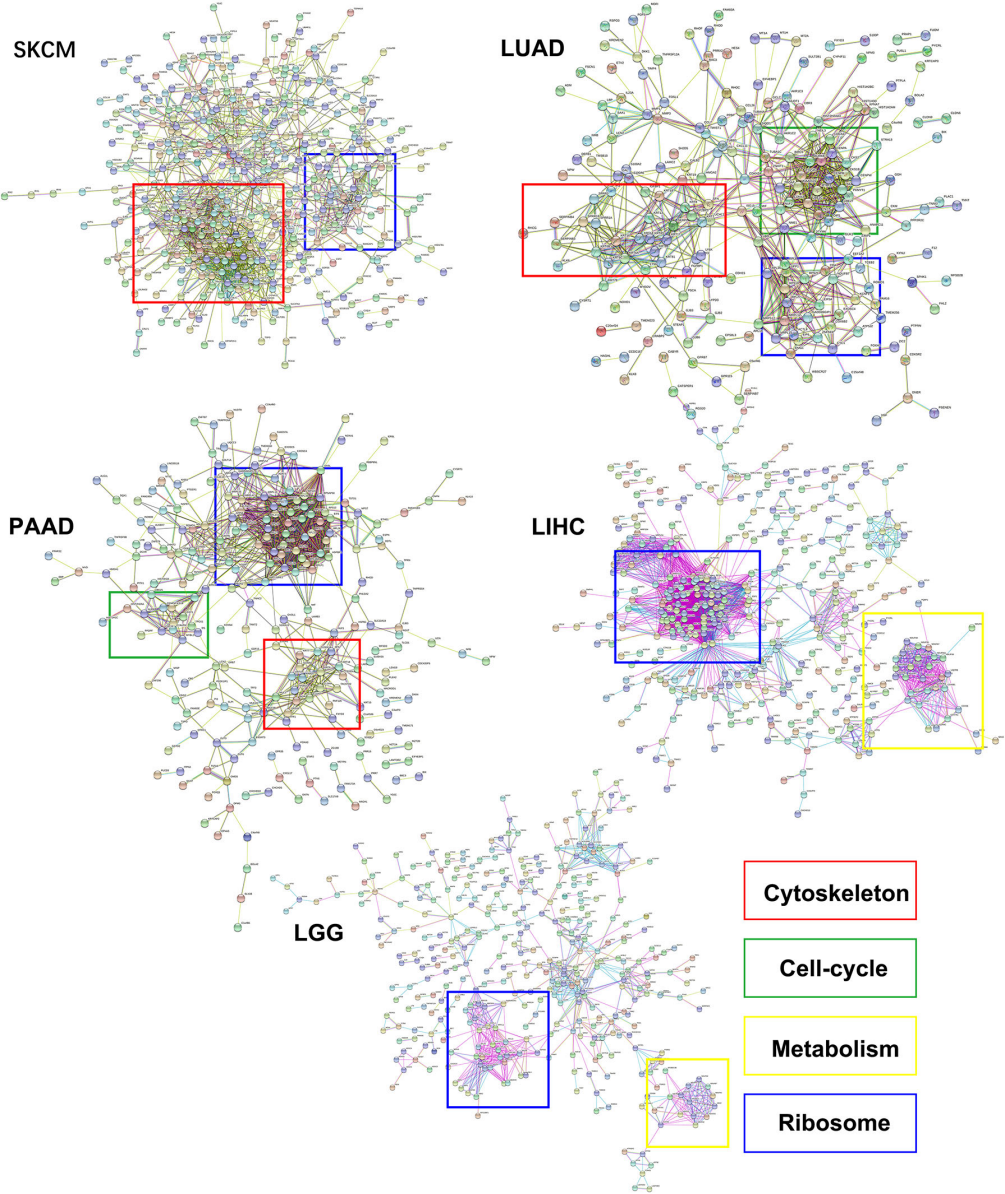
The protein-protein interaction clustering of differentially expressed genes while eIF6 was up-regulated in cancers. The significantly up-regulated genes in the eIF6 altered group were clustered according to protein interactions. The ribosomal proteins formed clusters in all five cancers. The cytoskeletal proteins were clustered in SKCM, LUAD, and PAAD, while the cell-cycle and metabolism proteins were clustered in PAAD and LUAD and LIHC cancers, respectively.

In this study, the genes with similar functions were converged together in the protein-protein interaction analysis map (**Figure 5**). Interestingly, the clustering results showed enrichment of keratin proteins in SKCM, LUAD, and PAAD, but not in LIHC and LGG. It is reasonable to assume that many types of cancers have a specific and well-coordinated genes modulation. This indicated that a more dynamic synthesis of intermediate filaments (IFs) occurred with tissue-specific regulation. Typically, the high intermediate filament protein levels were linked to cell proliferation and migration, requiring active cytoskeleton assembly and disassembly. For example, keratin17 was identified as a significantly up-regulated gene in eIF6-high melanoma, and this gene has been reported for its proliferation-promoting function in multiple cancers (31, 32). In fact, when we studied the up-regulated keratin protein effects on cancer progression, all the keratins we found were linked to lower survival rates in cancers, including melanoma (**Figure S4**). The all genes hazard ratio was above 1, with a *p* < 0.05 except KRTCAP2 (**Table 1**). This finding agreed with the fact that an up-regulated eIF6 could aggravate melanoma progression. Besides, we also identified the eIF6 co-expressed genes associated with RNA processing, metabolism and proliferation in the SKCM, which suggested the complexity of the eIF6 signal regulation network (**Figure S5**).

**Table 1:** Up-regulated keratin genes of the high-eIF6 expression group in SKCM, LUAD, PAAD, LGG, and LIHC.

	Differentially expressed genes between eIF6 altered and unaltered group					Survival analysis	
	Gene symbol	Cytoband	Log (fold change)	p-value	q-value	Hazard ratio	p(HR)
SKCM							
	KRT17	17q21.2	2.67	2.52E-04	6.33E-04	1.5	0.0018
	KRT6B	12q13.13	2.4	2.57E-03	5.40E-03	1.3	0.046
	KRT14	17q21.2	2.35	5.84E-03	0.0115	1.4	0.019
	KRT6A	12q13.13	2.3	7.22E-03	0.0139	1.5	0.0038
	KRT16	17q21.2	2.24	8.45E-03	0.0161	1.3	0.043
	KRT6C	12q13.13	2.12	8.84E-03	0.0167	1.4	0.017
	KRT5	12q13.13	1.98	0.0153	0.0275	1.3	0.036
	KRT1	12q13.13	1.82	0.0202	0.0355	1.3	0.05
	KRTAP19-1	21q22.11	1.7	6.29E-05	1.76E-04	1.7	0.00016
	KRTDAP	19q13.12	1.69	0.0123	0.0227	1.7	0.00015
	KRT15	17q21.2	1.6	2.34E-03	4.96E-03	1.3	0.045
	KRT75	12q13.13	1.54	3.73E-03	7.60E-03	/	/
	KRT19	17q21.2	1.45	7.63E-04	1.76E-03	1.3	0.082
LUAD							
	KRT6A	12q13.13	1.68	3.38E-05	1.99E-04	1.6	0.0033
	KRT16	17q21.2	1.51	2.67E-07	3.04E-06	1.6	0.0022
	KRT6B	12q13.13	1.28	2.09E-04	9.52E-04	1.7	0.00075
	KRT6C	12q13.13	1.27	7.19E-05	3.79E-04	1.8	0.00012
	KRT81	12q13.13	1.19	2.27E-05	1.42E-04	1.7	0.00037
	KRT17	17q21.2	1.08	6.47E-05	3.46E-04	1.6	0.0033
	KRT14	17q21.2	1.01	8.92E-04	3.34E-03	1.1	0.47
PAAD							
	KRT19	17q21.2	1.44	3.25E-08	1.43E-05	1.8	0.0045
	KRT15	17q21.2	1.34	1.69E-03	9.67E-03	1.4	0.091
	KRT18	12q13.13	1.09	2.04E-11	5.08E-08	1.7	0.011
	KRT7	12q13.13	1.07	6.05E-03	0.0239	2	0.001
	KRTCAP2	1q22	1.06	6.86E-05	1.32E-03	1.1	0.59
	KRT8	12q13.13	1.01	1.24E-06	1.38E-04	1.7	0.015
LGG							
	KRT18	12q13.13	1.15	9.89E-03	0.0215	2	0.00034
	KRT7	12q13.13	1.11	4.70E-03	0.0112	2	0.00023
	KRTCAP2	1q22	1.05	2.07E-08	3.48E-07	1.6	0.0075
LIHC							
	KRTCAP2	1q22	1.27	6.75E-11	2.92E-09	1.6	0.0086

This is the list of the keratin genes up-regulated over 2-fold (log fold change > 1) and changed with statistical significance (*p*-value < 0.05). The hazard ratio analysis allowed us to evaluate the role of these genes in the survival of patients with various cancers. All the listed genes hazard ratios were above 1-fold, which showed that their high expression were correlated with a worse prognosis.

### High eIF6 Expression Is Linked to the Global DNA Demethylation

**Figure 6:**
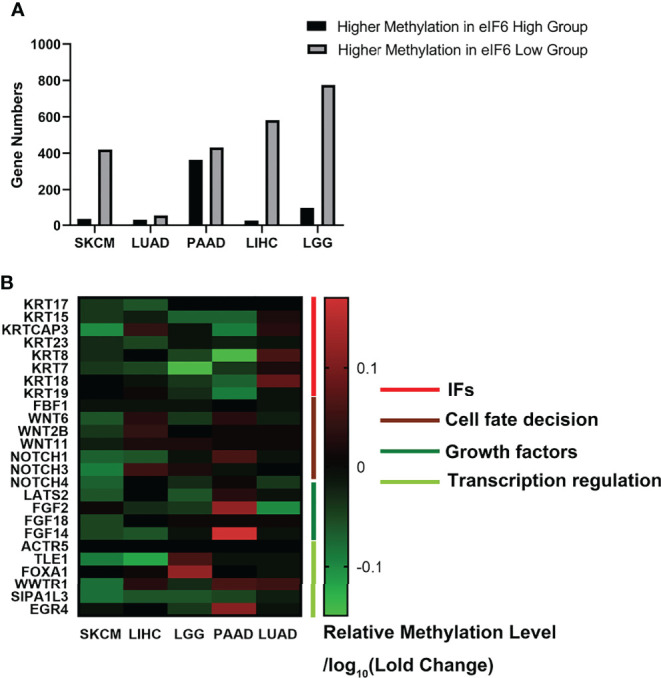
Genome methylation analysis in pan-cancer. **(A)** Comparison of the differentially methylated genes between high-eIF6 and low-eIF6 groups. In all the five cancers analyzed, the low-eIF6 groups had more genes with higher methylation levels than the high-eIF6 groups. **(B)** Heat map of differential methylated genes. The heat map was drawn according to the genes relative methylation level of the eIF6-high group compared to the eIF6-low groups. The demethylated genes were labeled with green. Upregulated keratins were identified as demethylated genes, including KRT17, KRT15, KRT23, and KRT7. Genes functioned in cell fate decisions, and cell growth was also found demethylated.

We further investigated the TCGA Firehouse Legacy sequencing data set. As previously stated, the specimens were grouped according to the level of eIF6 expression. The higher eIF6 expressed groups showed global genome demethylation in SKCM, LIHC, LGG, LUAD, and PAAD (**Figure 6A**). The methylation levels of the SKCM, LIHC, and LGG specimens were showed a significant difference between the eIF6-high and eIF6-low groups. In melanoma, there were 10305 genes had higher methylation levels in the eIF6-low group, while 5395 genes in the eIF6-high group had higher methylation tendencies. In the eIF6-high group, 420 genes were demethylated, and 35 genes were hypermethylated, which was significant larger than that in the eIF6-low group (**Table S2**).

We also clustered the demethylated genes in the five types of cancer, in which WNT and NOTCH family genes were found to be demethylated. These two gene clusters involved cell proliferation, differentiation, and cell fate (33). The methylation level of intermediate filament keratins was also investigated. The methylation levels of multiple keratin genes of the five cancers were decreased. In addition, KRT17 and KRT15 were up-regulated and demethylated in the eIF6-high expressed melanoma, suggesting that DNA demethylation was a potential transcription regulation mechanism of eIF6 (**Figure 6B**).

## Discussion

eIF6 has been reported as an essential regulator in liver hepatocellular carcinoma, colorectal carcinoma, and non-small cell lung carcinoma, respectively (21, 23, 24). Our study showed that the eIF6 up-regulation also occurs during melanoma tumorigenesis, which relates to a poorer prognosis. Furthermore, pan-cancer analysis revealed that the up-regulation of eIF6 is associated with demethylation and higher expression levels of intermediate filament keratins, which may account for the increased proliferation and migration rates in multiple types of cancer cells. This study offered a better understanding of the functional role of eIF6 in cancer progression and provided new insights into the potential role of eIF6 as a melanoma predictive biomarker.

Additionally, previous reports showed that both eIF4E and eIF2α are also closely associated with melanoma (34, 35). In this study, the skin melanoma samples exhibited significantly increased eIF6 expression levels than the normal skin samples, corresponding to the up-regulated melanoma cells division rates (**Figure 1**). Earlier studies have suggested that eIF6 is up-regulation in hepatocellular carcinoma, lung adenocarcinoma, and colorectal cancer (21, 23, 24). Similarly, the increased eIF6 expression levels also could be observed in LGG and PAAD using the GEPIA database analysis. Conversely, eIF6 was only significantly down-regulated in LAML (**Figure S1**). These findings revealed that eIF6 up-regulation is a common feature in different cancer groups, implying eIF6 was regulated by an cancer-or proliferation-related upstream regulators. The eIF6 promoter contains a GA-rich sequence, in which a GA binding protein (GABP) complex has been identified as an eIF6 expression modulator (36). In tumorigenesis, GABP is a well-studied transcription factor involved in regulating proliferation, ribosomes, and metabolism (37). Moreover, other tumor-related transcription factors, such as c-myb, can enhance the activation effect when combined with GABP complex (38). The increased expression of eIF6 in melanoma could be attributed to increased transcription factor binding caused by tumorigenesis. Therefore, the abnormal up-regulation of eIF6 in melanoma is a sign of cancer cell proliferation.

Additionally, the eIF6 level is also predictive of melanoma prognosis. GEPIA analysis revealed a landscape of thirty-three different types of cancers influenced by eIF6 (**Figure 2A**). Among the different types of cancer, high-eIF6 expressed patients had significantly lower survival rates than low-eIF6 expressed patients in SKCM, LGG, LIHC, LUAD, and PAAD. Subsequently, we intensively studied the survival rate of melanoma under different cut-off values (**Figure 2B**). Typically, the prognosis of the high-eIF6 expressed group was significantly poorer than that of the low-eIF6 expressed group. This was because cancer cells proliferate at a high rate, the protein translation requests were also upregulated. However, because eIF6 is a rate-limiting translation regulator, we hypothesized that elevated eIF6 levels aided cancer progression and thus resulted in a worse prognosis by limitation the protein synthesis. The *in vitro* experiments proved that eIF6 over-expressed A375 melanoma cells had a faster proliferation and migration rate (**Figures 3**, **4**). Indeed, a similar phenomenon has been previously observed in another melanoma cell line of WM793 (20). Therefore, it is reasonable to conclude that eIF6 is a critical regulator of tumor growth. Additionally, this hypothesis has also been proven because eIF6 knock-down could efficiently inhibit the progression of hepatocellular carcinoma and non-small cell lung carcinoma (21, 24). Since it has been reported that eIF6 is essential for immune system homeostasis in both mice and humans, we also investigated the tumor immunology of eIF6. Unexpectedly, all the results showed that no significant evidence suggested eIF6 could promote immune infiltration *via* immune modulation (**Figure S6–S10**) (39).

Besides acting as a nucleolus ribosomal genesis regulator, eIF6 can also regulate specific gene expression (40). We clustered the differential expression genes between high-eIF6 and low-eIF6 patients and found a co-expressive relationship between eIF6 and ribosomal proteins in SKCM, LIHC, LUAD, LGG, and PAAD (**Figure 5**). Since an abrogation of eIF6 hindered ribosomal 60S subunit biogenesis, the ribosomal proteins up-regulation also suggested that there may be a feedback loop involved in the regulation. In cancer cells, the higher ribosomal proteins expression and their corresponding protein translation may account for the lower survival rate of patients. In SKCM, LUAD, and PAAD clustering analysis, we found an up-regulation of keratin proteins in the high-eIF6 expression group (**Figure 5**). All the up-regulated keratins were correlated with the poor survival rate of patients (Hazard Ratio > 1; **Table 1**), which was consistent with the result of eIF6. In general, intermediate filaments (IFs), microtubes and microfilaments make up the cytoskeleton system of animal cells, in which, Keratin proteins are among the main components of IFs. There have been strong evidence indicating that the down-regulation or over-expression of IFs proteins can regulate various cellular behaviors, such as division, migration, growth, and apoptosis (41, 42). In this respect, eIF6 can selectively bind to IFs in mammalian cells, although the biological function of such complexes is not yet been determined, there is some evidence that the complexes formed by eIF6 and IFs are highly regulated during the oogenesis of Xenopus, suggesting they are probably contributing to the early development of embryo, which cells possessed the common feature of continuous mitosis with cancer cells (43). In order to understand this regulation mechanism better, a more detailed analysis is required in further studies. Nevertheless, as shown in **Table 1**, the high expression level of keratins show a dynamic assembly of IFs, contributing to cancer progression and metastasis, eventually leading to a lower patient survival rate.

Despite the detailed mechanisms of eIF6 that regulate keratins transcription remains unclear, eIF6 is commonly regarded as a vital translation regulator. It was reported that eIF6 could bind with chromosome DNA in the mitosis metaphase, which suggested that there may exist an unidentified mechanism that eIF6 directly regulates transcription (44). Herein we proposed a new concept that eIF6 may also regulate DNA methylation. We compared the number of genes whose methylation levels differed between the high-eIF6 and low-eIF6 groups (**Figure 6**). More higher-methylated genes were observed in the low-eIF6 group than that high-eIF6 group in all five types of cancers, suggesting eIF6 is an effective de-methylation regulator. With the up-regulated eIF6 levels, the lesser methylated whole genomes were well-matched with a more active genome transcription and more dynamic cellular activities, proliferation, and migration. In our study, the keratin genes up-regulated in high-eIF6 patients had a decreased methylation level (**Table 2**). We conclude that the IFs keratins are the downstream effectors of IF6, and the up-regulation of eIF6 causes a poor melanoma survival rate of patients by de-methylating and activating of keratin genes.

**Table 2:** The list of lesser methylated keratin proteins.

	Gene symble	Cytoband	p-Value	q-Value
SKCM				
	KRTAP19-1	21q22.11	0.0184	0.17
	KRT17	17q21.2	0.0212	0.18
	KRT15	17q21.2	0.0921	0.355
	KRT6B	12q13.13	0.138	0.431
LUAD				
	KRT16	17q21.2	0.0232	0.55
	KRT6B	12q13.13	0.244	0.823
PAAD				
	KRT8	12q13.13	1.93E-04	0.0181
	KRT18	12q13.13	1.40E-03	0.0408
	KRT19	17q21.2	3.01E-03	0.056
	KRT15	17q21.2	5.14E-03	0.0706
LGG				
	KRT7	12q13.13	9.98E-03	0.101

The methylation levels of the differentially expressed genes listed in **Table 1** were analyzed. A set of keratin genes was lesser methylated in the high-eIF6 group. The lower methylation level of keratins corresponded to their higher expression level in the high-eIF6 group.

## Conclusions

In summary, the up-regulated eIF6 could be a potential diagnostic and prognostic biomarker indicating poor survival of melanoma. We investigated the survival rate of 33 common types of cancers and found that the up-regulation of eIF6 was generally accompanied lower-survival rate. It is possible that eIF6 acts as a rate-limiting factor that induces higher dynamic cell skeleton gene expression and promotes the proliferation and migration of melanoma, which relates to a poorer prognosis. Herein we proposed that eIF6 is a promising biomarker to improve the assessment of clinical melanoma since the early clinical manifestations of melanoma often look like nevi. Considering the tremendous clinical value of eIF6, we believe that future medical applications will benefit patients.

## Data Availability Statement

The datasets presented in this study can be found in online repositories. The names of the repository/repositories and accession number(s) can be found in the article/**Supplementary Material**.

## Ethics Statement

The studies involving human participants were reviewed and approved by the Ethical Committee of the First Affiliated Hospital of Shenzhen University on 20210618. The ethical code is 20210615008. Written informed consent for participation was not required for this study in accordance with the national legislation and the institutional requirements.

## Author Contributions

FZ executed the experiments and analyzed the data. FZ and JW conceived the project design. FZ, SW, UA, and ZL co-wrote the manuscript. CZ, ZL, and JW supervised the work. All authors contributed to the discussion of results and commented on the final manuscript.

## Funding

This research was supported by National Natural Science Foundation of China (82172214), Guangdong Basic and Applied Basic Research Foundation (2020A1515010613, 2021A1515220176), The Key Basic Research Project of Shenzhen Science and Technology Program (JCYJ20200109115635440), Retired Expert Program of Guangdong Province (202020031911500002). Shenzhen-Hong Kong-Macau Technology Research Programme (Type C: SGDX2020110309300301).

## Conflict of Interest

The authors declare that the research was conducted in the absence of any commercial or financial relationships that could be construed as a potential conflict of interest.

## Publisher’s Note

All claims expressed in this article are solely those of the authors and do not necessarily represent those of their affiliated organizations, or those of the publisher, the editors and the reviewers. Any product that may be evaluated in this article, or claim that may be made by its manufacturer, is not guaranteed or endorsed by the publisher.

## Abbreviations

eIF6, Eukaryotic Initiation Factor 6; GEPIA, Gene Expression Profiling Interactive Analysis; IFs, Intermediate Filaments; KICH, Kidney Chromophobe; LAML, Acute Myeloid Leukemia; PCPG, Pheochromocytoma and Paraganglioma; LGG, Low-Grade Glioma; LIHC, Liver Hepatocellular Carcinoma; LUAD, Lung Adenocarcinoma; PAAD, Pancreatic Adenocarcinoma; GEPIA, Gene Expression Profiling Interactive Analysis; STRING, Search Tool for Recurring Instances of Neighboring Genes; SKCM, Skin Cutaneous Melanoma
