# Supplementary material for: eIF6 as a Promising Diagnostic and Prognostic Biomarker for Poorer Survival of Cutaneous Melanoma
Source: Front Oncol. 2022 May 30;12:848346. doi: 10.3389/fonc.2022.848346 (PMC9189357; doi:10.3389/fonc.2022.848346)
Supplement: Supplementary file 1 [file DataSheet_1.pdf]

Supplementary Information

eIF6 is a diagnostic and prognostic biomarker of poorer survival in cases of cutaneous melanoma

Zhang Fangyingnan[1], Saquib Waheed[2], Wu Jun[1,2], Zhang Chao[1], and Zhibin Li[2*]*

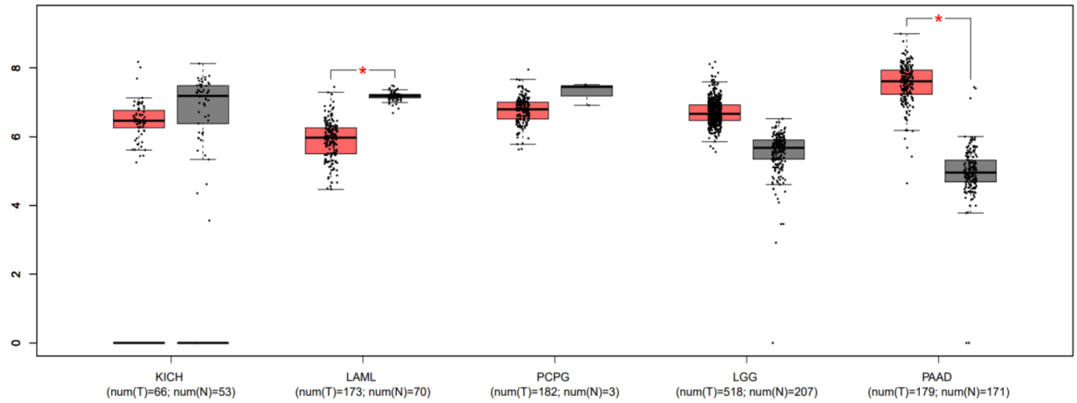

Figure S1. The GEPIA analysis of eIF6 expression in LGG, PAAD, KICH, LAMAL, and PCGP cancers.

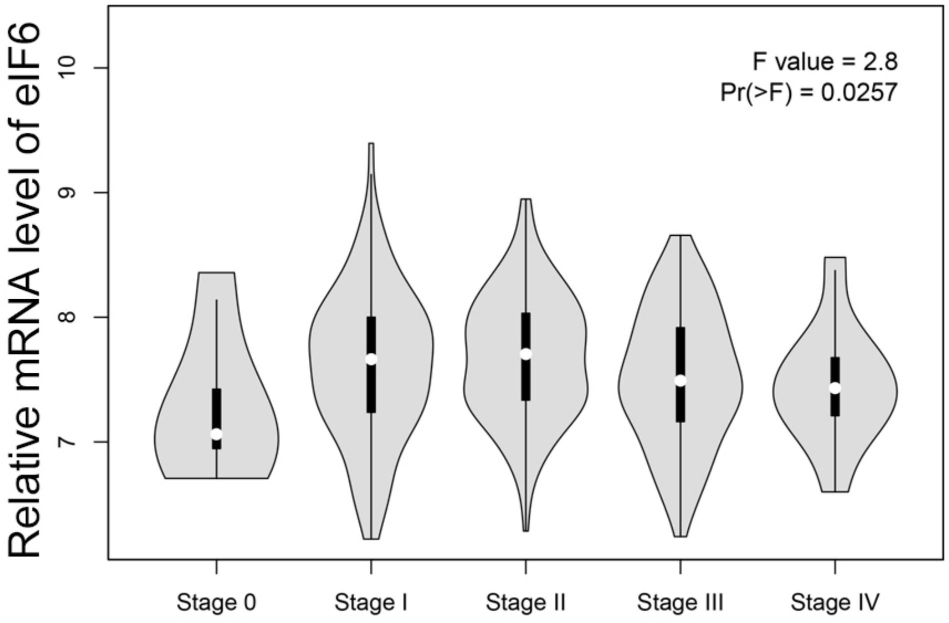

Figure S2. eIF6 level through all the stages of melanoma development. The eIF6 expression level was analyzed via GEPIA.

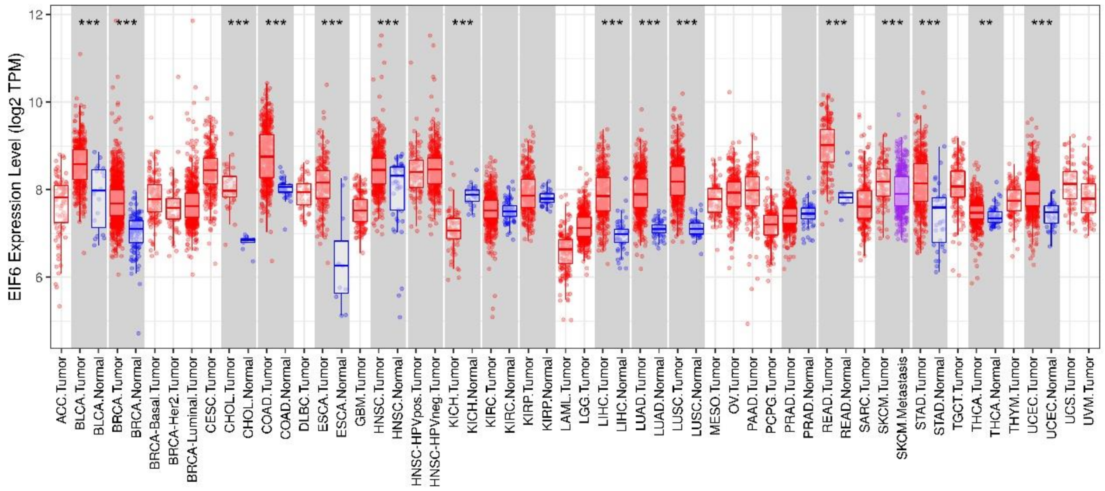

Figure S3. eIF6 level in skin melanoma and metastatic skin melanoma. The eIF6 expression level was analyzed via TIMER.

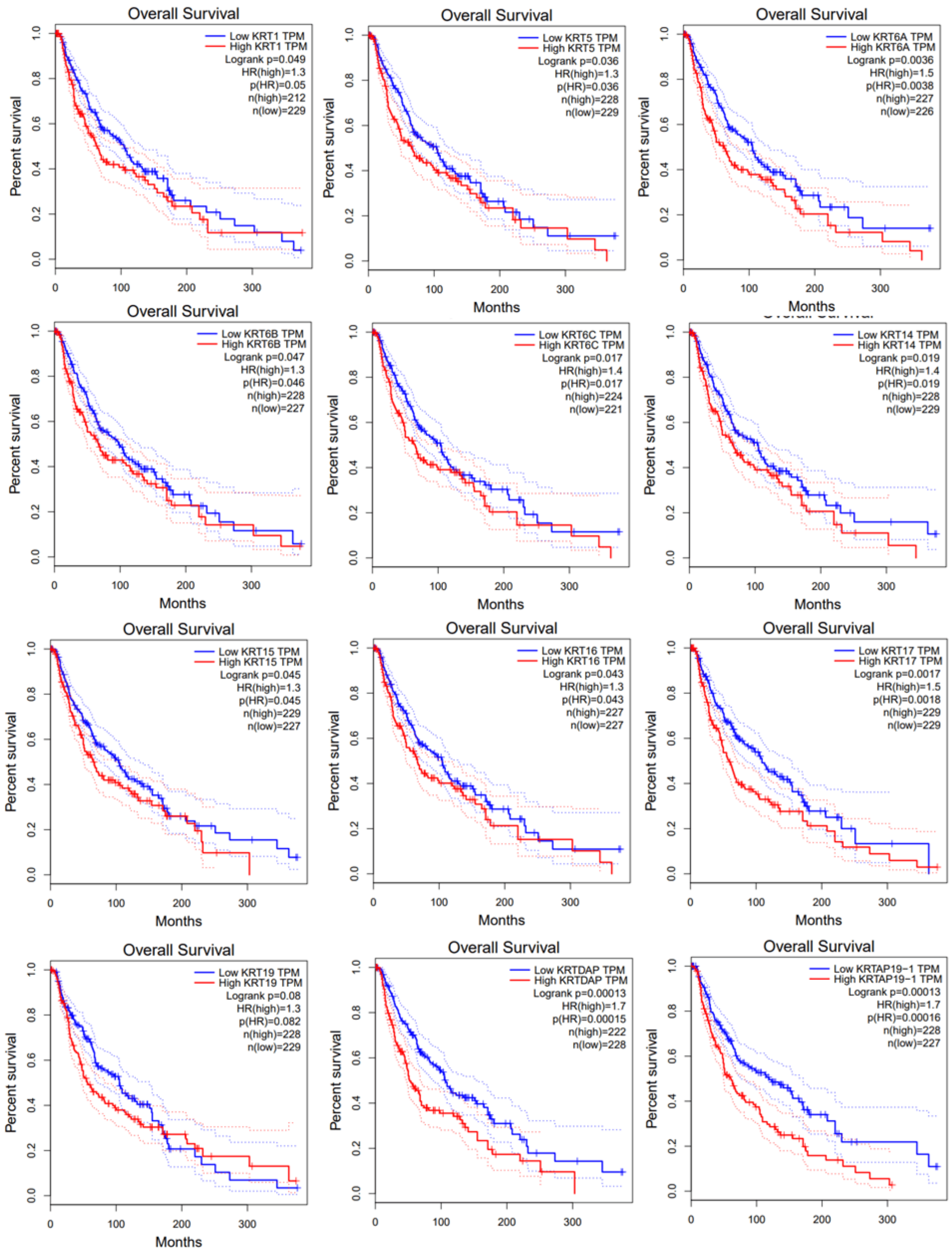

Figure S4. Survival analysis of eIF6 down-stream genes.

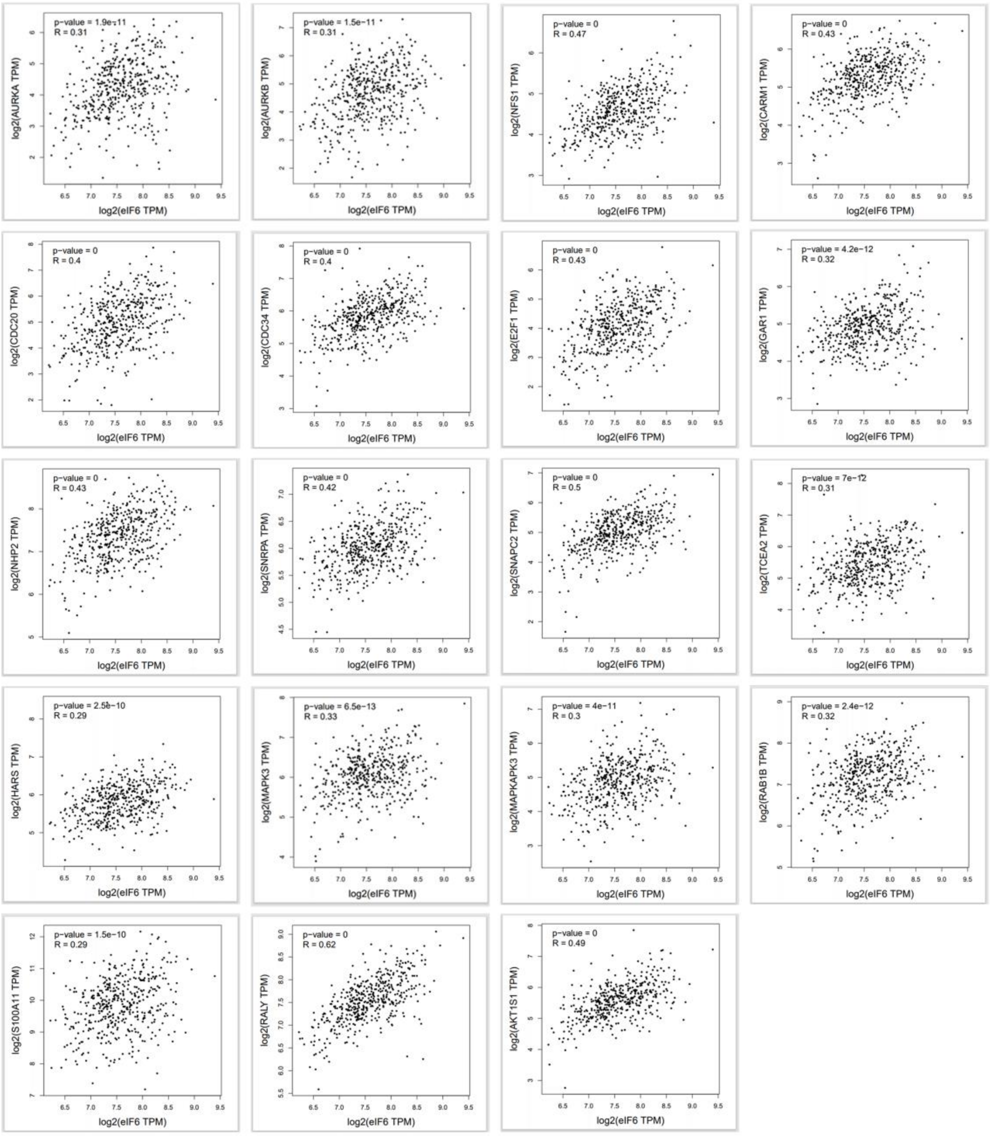

Figure S5. Co-expression analysis of eIF6 in SKCM.

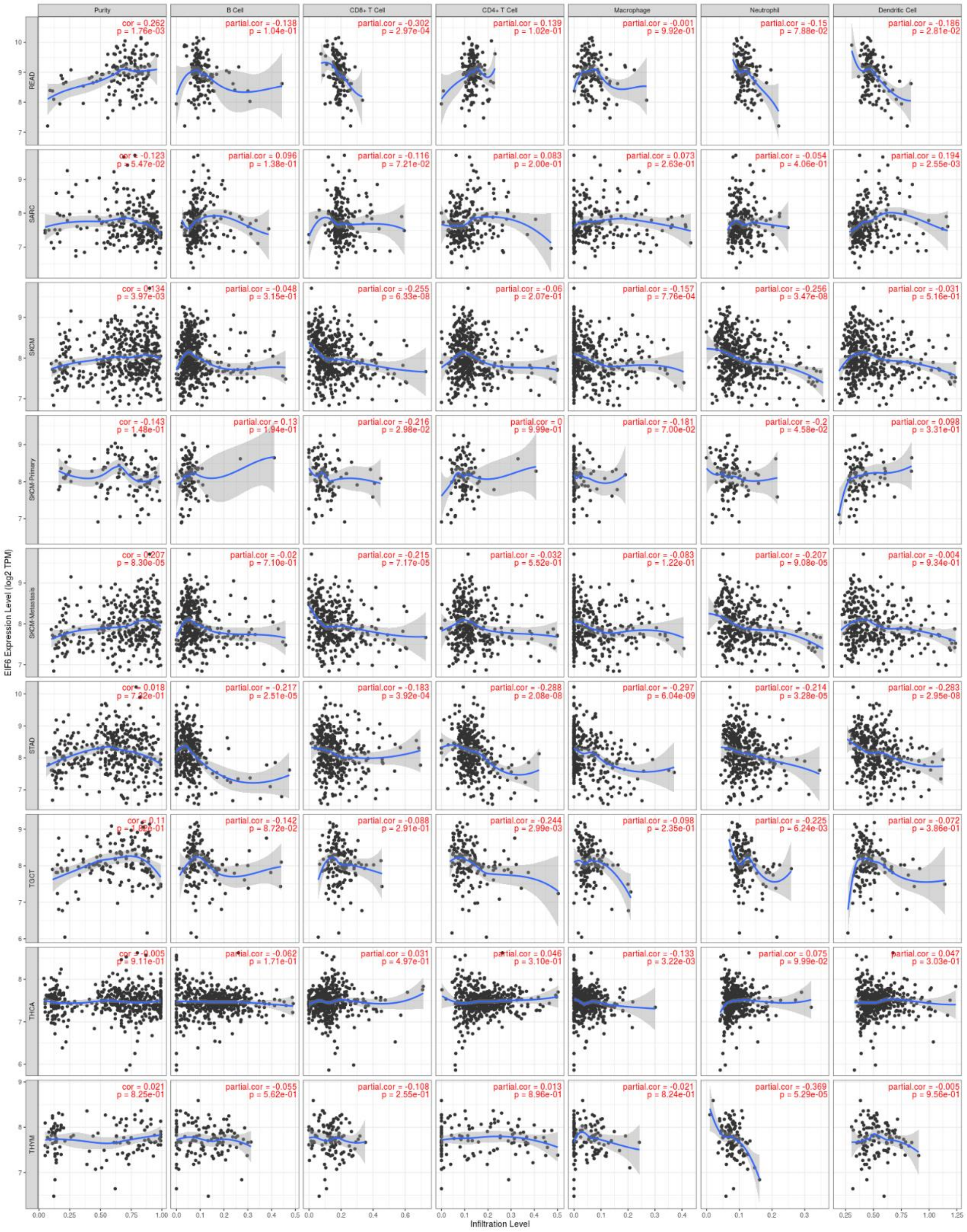

Figure S6. Correlation analysis between immune infiltration and eIF6 level in READ, SARC, SKCM, STAD, TGCT, THCA and THYM.

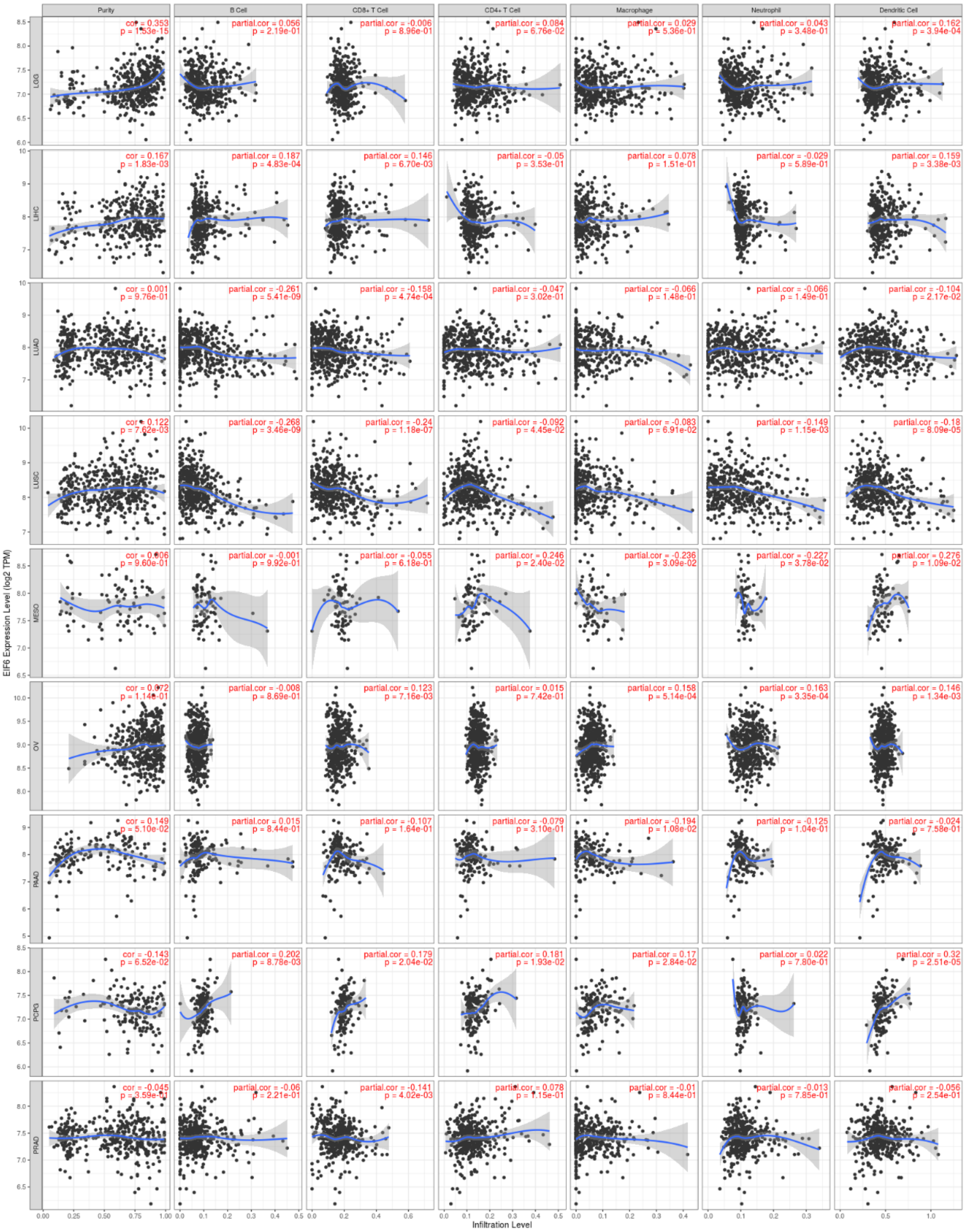
Purity
B Cell
CD8+ T Cell
CD4+ T Cell
Macrophage
Neutrophil
Dendritic Cell
LGG
cor = 0.353 p = 1.53e-15
partial.cor = 0.056 p = 2.19e-01
partial.cor = -0.006 p = 8.96e-01
partial.cor = 0.084 p = 6.76e-02
partial.cor = 0.029 p = 5.36e-01
partial.cor = 0.043 p = 3.48e-01
partial.cor = 0.162 p = 3.94e-04
LIHC
cor = 0.167 p = 1.83e-03
partial.cor = 0.187 p = 4.83e-04
partial.cor = 0.146 p = 6.70e-03
partial.cor = -0.05 p = 3.53e-01
partial.cor = 0.078 p = 1.51e-01
partial.cor = -0.029 p = 5.89e-01
partial.cor = 0.159 p = 3.38e-03
LUAD
cor = 0.001 p = 9.76e-01
partial.cor = -0.261 p = 5.41e-09
partial.cor = -0.158 p = 4.74e-04
partial.cor = -0.047 p = 3.02e-01
partial.cor = -0.066 p = 1.48e-01
partial.cor = -0.066 p = 1.49e-01
partial.cor = -0.104 p = 2.17e-02
LUSC
cor = 0.122 p = 7.82e-03
partial.cor = -0.268 p = 3.46e-09
partial.cor = -0.24 p = 1.18e-07
partial.cor = -0.092 p = 4.45e-02
partial.cor = -0.083 p = 6.91e-02
partial.cor = -0.149 p = 1.15e-03
partial.cor = -0.18 p = 8.09e-05
MESO
cor = 0.006 p = 9.60e-01
partial.cor = -0.001 p = 9.92e-01
partial.cor = -0.055 p = 6.18e-01
partial.cor = 0.246 p = 2.40e-02
partial.cor = -0.236 p = 3.09e-02
partial.cor = -0.227 p = 3.78e-02
partial.cor = 0.276 p = 1.09e-02
OV
cor = 0.072 p = 1.14e-01
partial.cor = -0.008 p = 8.69e-01
partial.cor = 0.123 p = 7.16e-03
partial.cor = 0.015 p = 7.42e-01
partial.cor = 0.158 p = 5.14e-04
partial.cor = 0.163 p = 3.35e-04
partial.cor = 0.146 p = 1.34e-03
PAAD
cor = 0.149 p = 5.10e-02
partial.cor = 0.015 p = 8.44e-01
partial.cor = -0.107 p = 1.64e-01
partial.cor = -0.079 p = 3.10e-01
partial.cor = -0.194 p = 1.08e-02
partial.cor = -0.125 p = 1.04e-01
partial.cor = -0.024 p = 7.58e-01
PCPG
cor = -0.143 p = 6.52e-02
partial.cor = 0.202 p = 8.78e-03
partial.cor = 0.179 p = 2.04e-02
partial.cor = 0.181 p = 1.93e-02
partial.cor = 0.17 p = 2.84e-02
partial.cor = 0.022 p = 7.80e-01
partial.cor = 0.32 p = 2.51e-05
PRAD
cor = -0.045 p = 3.59e-01
partial.cor = -0.06 p = 2.21e-01
partial.cor = -0.141 p = 4.02e-03
partial.cor = 0.078 p = 1.15e-01
partial.cor = -0.01 p = 8.44e-01
partial.cor = -0.013 p = 7.85e-01
partial.cor = -0.056 p = 2.54e-01
EIF6 Expression Level (log2 TPM)
Infiltration Level

Figure S7. Correlation analysis between immune infiltration and eIF6 level in LGG, LIHC, LUAD, LUSC, MESO, OV, PAAD, PCPG and PRAD.

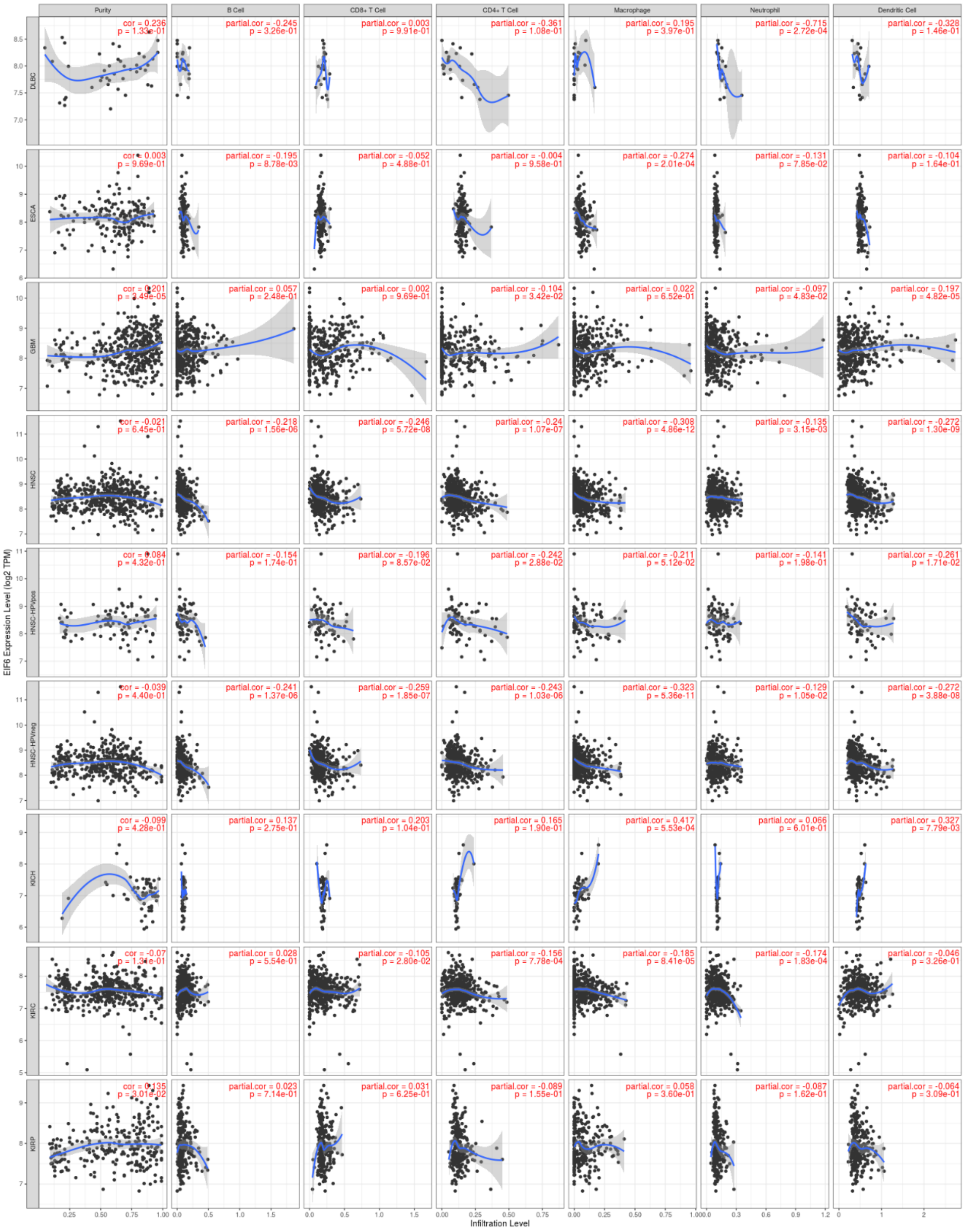

Figure S8. Correlation analysis between immune infiltration and eIF6 level in DLBC, ESCA, GBM, HNSC, KICH, KIRC and KIRP.

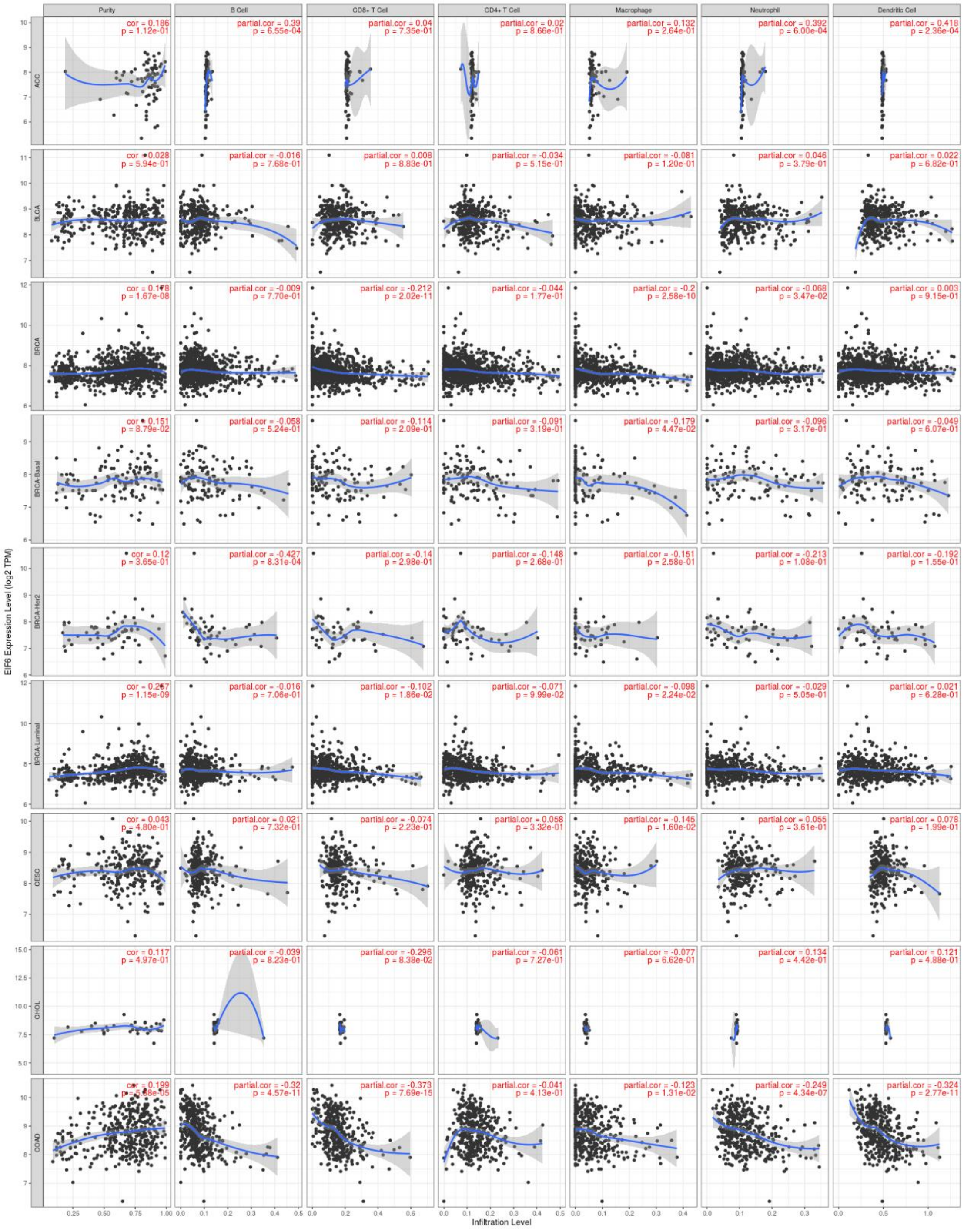

Figure S9. Correlation analysis between immune infiltration and eIF6 level in ACC, BLCA, BRCA, CESC, CHOL and COAD.

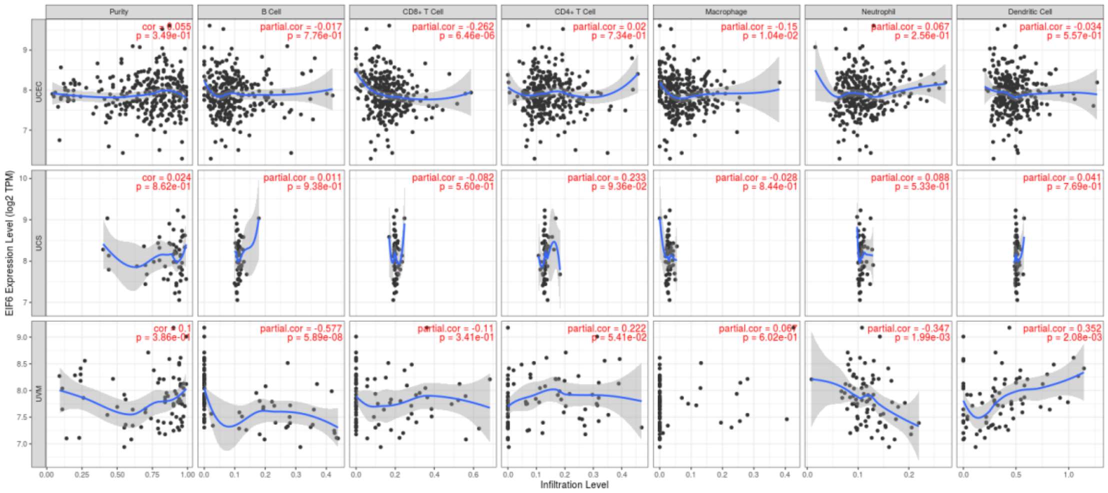

Figure S10. Correlation analysis between immune infiltration and eIF6 level in UCEC, UCS and UVM.
